# Supplementary material for: Aspartate aminotransferase Rv3722c governs aspartate-dependent nitrogen metabolism in Mycobacterium tuberculosis
Source: Nat Commun. 2020 Apr 23;11:1960. doi: 10.1038/s41467-020-15876-8 (PMC7181641; doi:10.1038/s41467-020-15876-8)
Supplement: Supplementary file 1 — Supplementary Information [file 41467_2020_15876_MOESM1_ESM.pdf]

Supplementary Information

Aspartate aminotransferase Rv3722c governs aspartate-dependent nitrogen metabolism in *Mycobacterium tuberculosis*

Jansen *et al*.

Supplementary Notes

Supplementary Note 1

The X-ray diffraction data of Rv3722c in complex with Glu was initially reduced in the tetragonal crystal system and was found to belong to the $P4_22_12$ space group. Molecular replacement using the ligand free structure of the same enzyme (PDB 5C6U) as a search model, yielded a solution comprising of a dimer in the asymmetric unit. Attempts, however, to refine the solution were unsuccessful, as the R_{free} stalled above 40%. Analysis of the data in Xtriage in Phenix[1], revealed that the intensity statistics significantly deviated from those expected for a good to reasonable and untwinned data. In addition, the Patterson function revealed the presence of an off-origin peak at fractional coordinates (0.500 0.500 0.388), with a height approximately 45% of the origin. It has been reported that crystal pathologies such as twinning or pseudo-symmetry, can lead to wrong space group assignment because the data have an apparent high symmetry than its actual symmetry[2 3]. Indeed, when the data was re-indexed in the lower symmetry P222 point group, the space group was found to be $P2_12_12$. The structure was solved by molecular replacement as described above. The resulting solution had four molecules (two dimers) in the asymmetric unit. Subsequent analysis of the data in Xtriage revealed it displayed both pseudo-merohedral twinning and pseudo translation. As a result, the twin law (-h, l, k) was used in the final round of refinement.

Supplementary Note 2

For the structure of Rv3722c/Glu complex, residues 3 – 422 could be built into the electron density and was refined with R_{work} equal to 19.3 % and R_{free} equal to 21.6 % (Supplementary Table 1). On the other hand, Rv3722c after pre-incubation with L-kynurenine crystallized in the trigonal $P3_1$ space group and diffracted to 2.23 Å. There were eight molecules in the asymmetric unit. Residues 2 – 430 could be built into electron density and was refined with R_{work} equal to 17.9 % and R_{free} equal to 21.9 % (Supplementary Table 1).

The overall fold of Rv3722c is similar to that of members of the recently founded Type Ic group of PLP-binding proteins[4]. Each monomer consists of a core domain (Pro54 - Gly302) that folds into a nearly perfect α/β motif comprised of a central eight -stranded (predominantly antiparallel) β- sheet surrounded by eight α- helices. The auxiliary domain (Ser2 - Leu53 and Asp303 – Ser430), on the other hand, stacks and forms an elongated segment on top of the core domain and consists of five α – helices and an antiparallel β- sheet. A VAST search[5] showed that the overall structure of Rv3722c was similar to the aspartate transaminase from *Corynebacterium glutamicum* (PDB 5IWQ)[4]. The root mean square difference across 418 Cα atom pairs is 1.1 Å. The two enzymes also share a high degree of sequence homology with 55% identity and 72% similarity.

Supplementary Note 3

From the structure of the Glu pre-incubated protein we observed positive electron density in the active sites of the molecules in the asymmetric unit. We observed clear electron density of Glu along that of PLP in chain B (Fig 4A and Supplementary Figure 7). The rest of the chains were only bound to PLP.

Glu binds in the active site pocket located in the convergence of the auxiliary and core domain. The amino group of Glu is in close proximity to the aldehyde group of PLP at a distance of 2.8 Å. The α-carboxylate of Glu forms hydrogen bonds with (NH1; 2.90 Å) and (NH2; 2.8 Å) groups of the guanidinium side chain of Arg392. The carboxylate side chain of Asp140, whose protonation state is unclear, is within range (3.6 Å) to interact with the α-carboxylate group of Glu. AspATs coordinate side chain carboxylate groups of dicarboxylic substrates through a conserved active site arginine (or sometimes a lysine)[6,7]. In Rv3722c the corresponding residue is Arg141 and it forms salt bridges with the side chain carboxylate group Glu (NH2; 4.40 Å). Hydrophobic contacts with Tyr69* and Ile287* (asterisk denotes a residue from the second monomer) further stabilize Glu in the binding pocket.

Supplementary Note 4

We observed different ligand bound states in the molecules making the asymmetric unit of the crystals of Rv3722c pre-incubated with L-kynurenine (Kyn) (Supplementary Figure 7 and 8). For accurate ligand modelling, Polder omit maps were generated[9]. The active site pocket of chain A is bound to the external aldimine intermediate PLP-kynurenine (PLP-Kyn) while the keto acid product, kynurenic acid (Kyna), was bound in chains B, C, D and G. In chains E, F and H, however, the map in the binding pocket was deemed uninterpretable and was left unmodeled.

Compared to the ligand-free structure of Rv3722c, the binding of Kyn induced only the rearrangement of the sidechain of Arg36 in the active site pocket (Supplementary Figure 10). In the unbound form of Rv3722c, the positively charged guanidinium side chain of Arg36 points outwardly and lies above the entrance of the active site pocket. However, upon binding Kyn, the Cα of Arg36 shifts by ~ 1 Å and the guanidinium side chain is moved inwardly by ~ 12 Å (measured from the Cζ atom) to interact with Kyn (Supplementary Figure 10). This ligand-induced conformation change is reminiscent of, but serves a totally opposite function than, the so called "arginine switch" observed in Type Ia AspAT[10]. An overlay of the Kyn-bound structure with that of the ligand free structure of Rv3722c, shows that Arg36 is not in a position to sterically clash with the bulky side chain of Kyn (Supplementary Figure 10). This observation suggests that the ligand-induced movement is necessary for the recognition and stabilization of the aromatic ligand. Apart from interacting with Kyn, Arg36 is locked in position by interacting with Asp140 through a bidentate salt bridge (NH1 and Oδ2; 3.6 Å) and (NH2 and Oδ1; 3 Å).

In addition to being linked to PLP, the α-carboxylate group of Kyn forms a hydrogen bond (NH1; 2.9 Å) and a salt bridge (NH2; 3.5 Å) with Arg392. The carbonyl oxygen of Kyn is hydrogen bonded to (NE; 3.1 Å) Arg36. The carbonyl group of Kyn is also hydrogen bonded (NE; 3.1 Å) to the side chain of Arg141. The arene ring of Kyn forms hydrophobic contacts with Tyr69* and Ile287*.

In the first half reaction of transamination, the active site lysine residue abstracts a proton from the Cα of the external aldimine yielding a quinonoid intermediate[11]. Further reprotonation of the cofactor by the same lysine residue generates the ketimine intermediate, which is subsequently hydrolyzed to release the keto acid of the amino donor while generating PMP. In our structure, we observed a sharp peak of a water molecule in close proximity to both the active site Lys257 residue and PLP-KYN external aldimine (Supplementary Figure 8A). It is tempting to speculate that this is the water molecule responsible to hydrolyze the PLP-KYN intermediate, generating Kyna.

Interestingly, the product Kyna adopts a different pose relative to its precursor (Supplementary Figure 8). In comparison to Kyn, the carboxylate group of Kyna is rotated by ~ 180° and faces the entrance of the solvent-exposed binding pocket (Figure 4C). Unlike Kyn, the product is engaged mainly by non-polar interactions in the binding pocket. The hydroxyl group forms a hydrogen bond with (NE; 2.6 Å) Arg141. Interestingly, in the product bound chains, Arg36 adopts its original "outward" conformation, most likely facilitating the release of the product (Figure 4C and Supplementary Figure 8). Additional hydrophobic contacts with Tyr139, Tyr69*, Ile287* stabilize Kyna in the binding pocket.

Supplementary Note 5

ABMP and enzyme kinetic studies indicated that Rv3722c had significant side activity for the aromatic metabolite L-kynurenine. To our surprise, none of the aromatic amino acids were preferred by Rv3722c as amino donors. The crystal structure of Rv3722c bound to L-kynurenine provide clues why it is a suitable amino donor over the other aromatic ligands. Firstly, it appears that Arg141 is strategically positioned to demarcate the binding pocket and restrict the pose adopted by ligands in the pocket. Indeed, the binding pose adopted by Kyn could explain why it a preferred substrate. The aliphatic backbone of Kyn mimics the dicarboxylic amino acid Asp (Fig 4D). Superimposing Asp into the electron

density of the aliphatic chain of Kyn reveals that not only are the amino groups and α-carboxylates superimposable, but also importantly, the carbonyl oxygen of Kyn is at an analogous position as the O6 atom of the carboxyl side chain of Asp (Fig 4D). As a result, this allows it form hydrogen bonds with Arg141 and Arg36. In addition, this binding pose allows the aromatic ring to form hydrophobic contacts with Tyr69* and Ile287*– further stabilizing the ligand prior to transamination. The presence of the carbonyl group in Kyn and the lack thereof in aromatic amino acid substrates such as Trp, Phe and Tyr, appears to be one of the selectivity factors for binding in the active site pocket as well as the capacity to serve as an amino donor. Taken together, these data provide insights as to why, in addition to its cognate dicarboxylic acid substrates, Rv3722c is able to bind in the same pocket and utilize as a substrate, the structurally disparate aromatic ligand Kyn. Lastly, the high degree of residue conservation in the active sites of Type Ic AspATs, suggests that side activity towards Kyn could be a hallmark of these enzymes. However, the physiologic relevance of this side activity, especially in the context of *Mycobacterium tuberculosis*, remains unclear and worth exploring.

Supplementary Tables

Supplementary Table 1. Data collection and refinement statistics.

	Rv3722c/Glu	Rv3722c/KYN
**Data collection**		
Space group	P 2_1 2_1 2	P 3_1
Cell dimensions		
a, *b*, *c* (Å)	87.784 173.691 173.515	108.398 108.398 321.344
α, β, γ (°)	90 90 90	90 90 120
Resolution (Å)	48.33 - 2.60 (2.693 - 2.60)	48.36 - 2.237 (2.342 - 2.237) *
R_{sym} or R_{merge}	0.148 (0.564)	0.138 (0.692)
$I / \sigma I$	9.4 (3.2)	
Completeness (spherical %)	99.76 (99.36)	87 (29.9)
Completeness (ellipsoidal %)		89.5 (34.2)
Redundancy	7.5 (6.6)	7.5 (4.2)
**Refinement**		
Resolution (Å)	48.33 - 2.60	48.36 - 2.237
No. reflections	82130 (8067)	178462 (5059)
R_{work} / R_{free}	0.193 / 0.216	0.1792 / 0.2193
No. atoms	13087	27421
Protein	12921	26019
Ligand/ion	58	357
Water	108	1045
B-factors		
Protein	31.82	36.98
Ligand/ion	29.65	41.30
Water	17.16	33.25
R.m.s. deviations		
Bond lengths (Å)	0.003	0.010
Bond angles (°)	0.64	1.28

Statistics for the highest-resolution shell are shown in parentheses.
Asterisk: Resolution limit after anisotropy correction using STARANISO.

Supplementary Figures

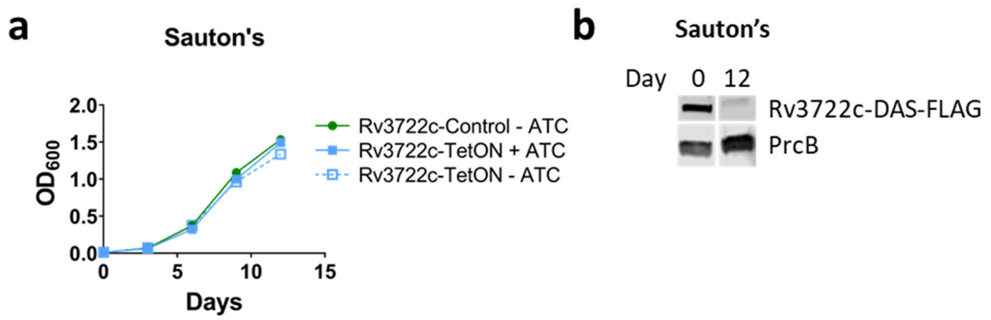

Supplementary Figure 1. Rv3722c is dispensible in defined Sauton's minimal media **A)** Growth curve of Rv3722c-proficient and –deficient *Mtb* in defined Sauton' s minimal media. Rv3722c-TetOn and Rv3722c-control cultured in Middlebrook 7H9 culture media with 500 ng mL^{-1} anhydrotetracycline (ATC) were used to inoculate Sauton's minimal media with and without 500 ng mL^{-1} ATC. Bacterial growth was monitored for 12 days, by optical density at 600 nm. Data are represented as mean -/+ SD of three experimental replicates (n=3), representative of at least two independent experiments. **B)** Western blot showing depletion of Rv3722c after 12 days of culturing in Sauton's without ATC (A). Protein lysates were analyzed by Western blotting, using an α-FLAG antibody. The proteasome subunit β (PrcB) was used as loading control. Depletion of Rv3722c below the lower limit of detection has been independently reproduced in three experiments. Source data are provided as a Source Data file.

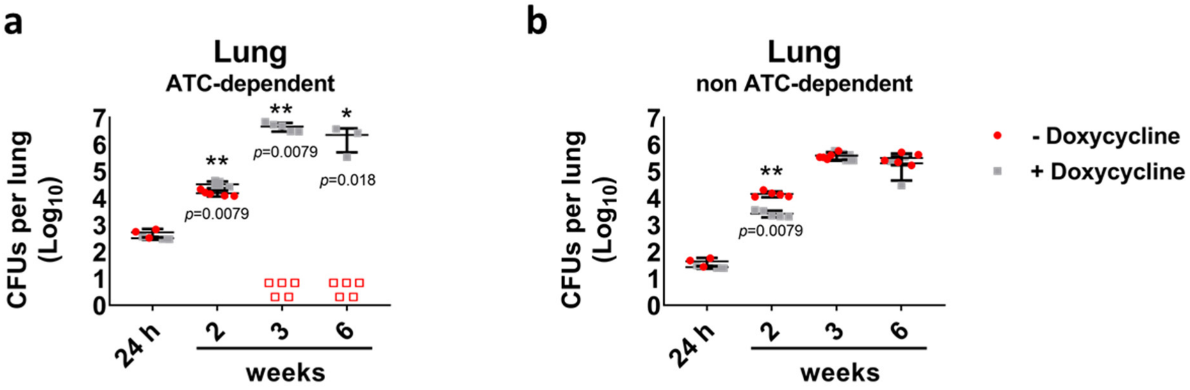

Supplementary Figure 2. Rv3722c is required for virulence in mice. Mice were infected with aerosolized Rv3722c-TetON pre-cultured in Sauton's with and without ATC to generate Rv3722c-proficient and -deficient *Mtb*, respectively. After infection, mice were fed chow with (Rv3722c-proficient *Mtb*) or without (Rv3722-deficient *Mtb*) doxycycline. The number of CFUs were assessed by plating serial dilutions on 7H10 solid media with ATC. The number of ATC-dependent bacteria (**A**) and non ATC-dependent mutants (**B**) were determined by plating on 7H10 solid media with and without ATC (n=2). Data are represented as mean +/- SD of five mice (n=5), except for the 24h timepoints (n=3) and the 6 week + doxycycline timepoint (n=3). Open symbols indicate samples in which the bacterial burden was below the limit of detection (less than 2 CFUs). Within 3 weeks after aerosol infection of mice, Rv3722-deficient *Mtb* were almost completely replaced by escape mutants that were no longer ATC-dependent. Spleen data are not shown because the number of non ATC-dependent mutants was not determined. Statistically significant differences were identified by unpaired, 2-sided Mann-Whitney *U* rank testing (single asterisk: $p< 0.05$, double asterisk: $p<0.01$, as indicated). Source data are provided as a Source Data file.

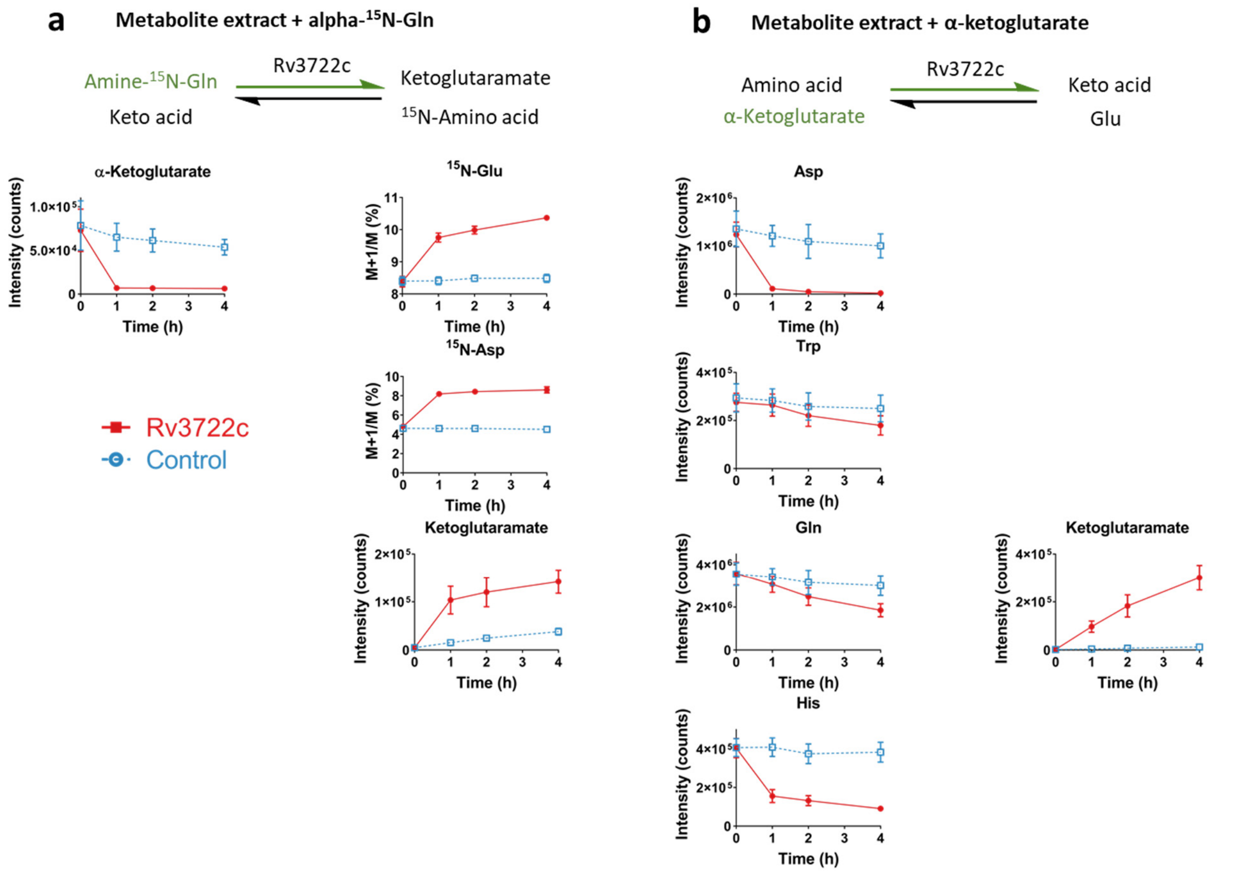

Supplementary Figure 3. Rv3722c functions as an aminotransferase. **A)** Activity-based metabolite profiling (ABMP) with Rv3722c in the presence of α-^{15}N-Gln. Purified recombinant Rv3722c (10 µM; red line) or a heat-inactivated control (10 min 95 °C; blue line), was incubated with a mycobacterial metabolite extract supplemented with 10 mM α-^{15}N-Gln for 0, 1, 2 and 4 h at 37 °C, and analyzed using untargeted LC-MS. **B)** Activity-based metabolite profiling (ABMP) with Rv3722c in the presence of α-ketoglutarate. Same as A, but using a mycobacterial metabolite extract supplemented with 20 mM α-ketoglutarate. Colored arrows indicate the forced direction of the Rv3722c-mediated reaction. Relative metabolite levels are represented as intensity, while ^{15}N-labeling is presented as the ratio M+1/M, which was not corrected for naturally occuring isotopes. Data are presented as mean +/- SD of three experimental replicates (n=3). Source data are provided as a Source Data file.

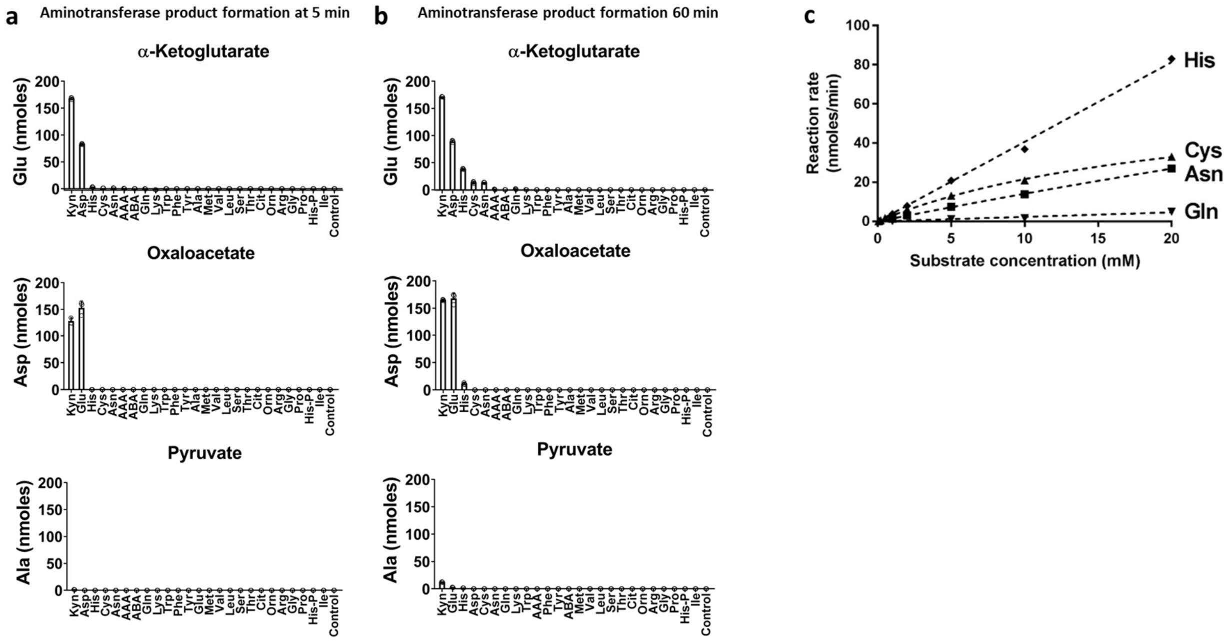

Supplementary Figure 4. Rv3722c substrate screen. **A)** Rv3722c product formation after 5 min incubation. Purified recombinant Rv3722c (1 µM) was incubated with a panel of amino acids (1 mM) and 3 common keto acids (α-ketoglutarate, oxaloacetate or pyruvate; 10 mM). After 5 minutes at 37°C, product formation (Glu, Asp or Ala for α-ketoglutarate oxaloacetate or pyruvate, respectively) was measured by RapidFire mass spectrometry. **B)** Rv3722c product formation after 60 min incubation. Same as A, but after incubating for 60 minutes. Data are presented as individual values (open circles) and mean +/- SD (n=3). AAA: aminoadipic acid; ABA: 2-aminobutyric acid; Cit: citrulline; Orn: ornithine; His-P: histidinol phosphate. **C)** Steady-state enzyme kinetics of Rv3722c for His, Cys, Asn and Gln. Purified recombinant Rv3722c (1 µM) was incubated with 10 mM α-ketoglutarate and increasing concentrations of amino donors at 37 ^{o}C. Glu formation was measured by RapidFire mass spectrometry and used to determine initial reaction rates. Data were fitted to Michaelis-Menten kinetics using Graphpad Prism software and are represented as mean +/- SD of three experimental replicates (n=3). Source data are provided as a Source Data file.

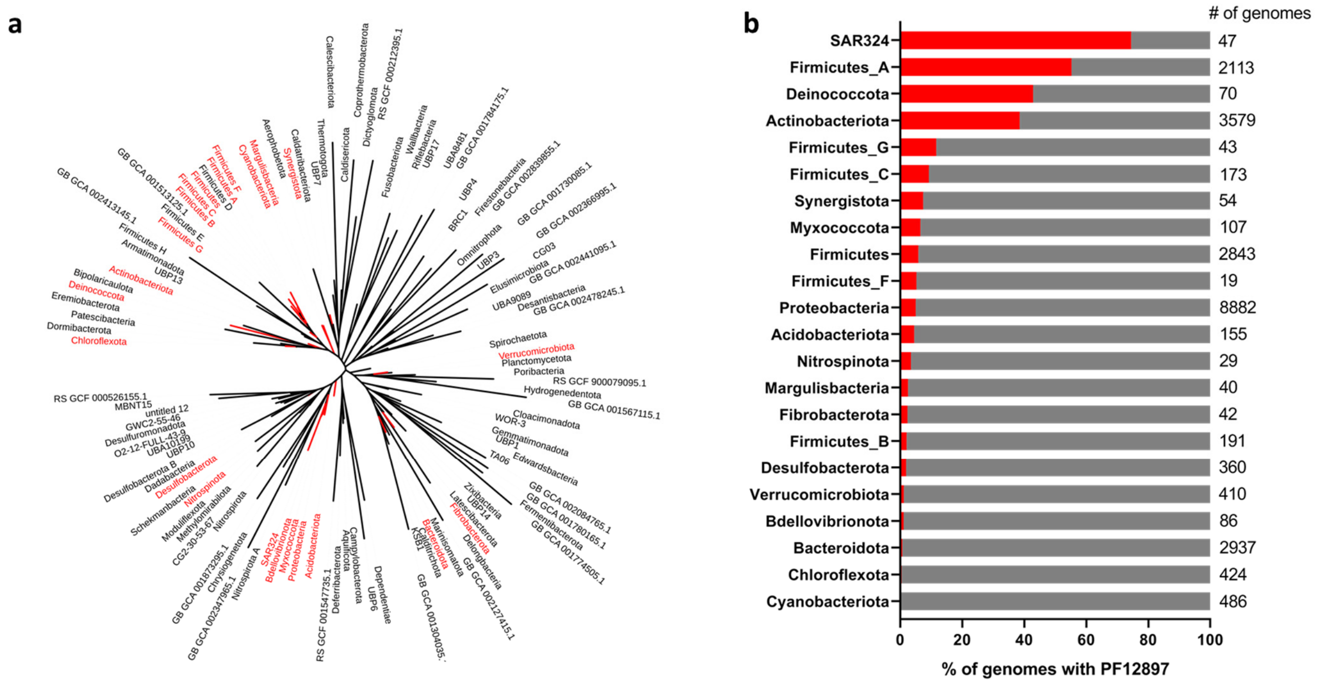

Supplementary Figure 5. PF12897 family members are present in selective bacterial phyla. **A)** Phylogenetic tree showing the distribution of PF12897 across bacterial phyla. Phyla highlighted in red contain at least 1 genome containing a PF12897 family member. **B)** PF12897 frequency and size of phyla containing PF12897 family members. Source data are provided as a Source Data file.

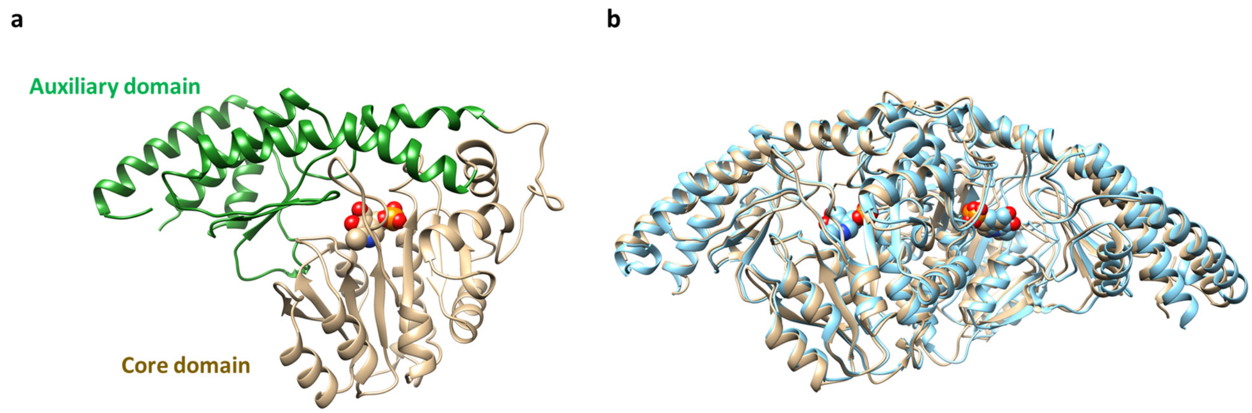

Supplementary Figure 6. Rv3722c is a type Ic PLP-binding protein. **A)** Ribbon representation of the monomer of Rv3722c showing the domain architecture of the enzyme. The auxiliary domain is colored in green and the core domain in tan. **B)** Ribbon representation of the superposition of Rv3722c (tan) with the type Ic AspAT from *Corynebacterium glutamicum* (5IWQ; cyan). The cofactor PLP is shown as spheres in the active site pocket.

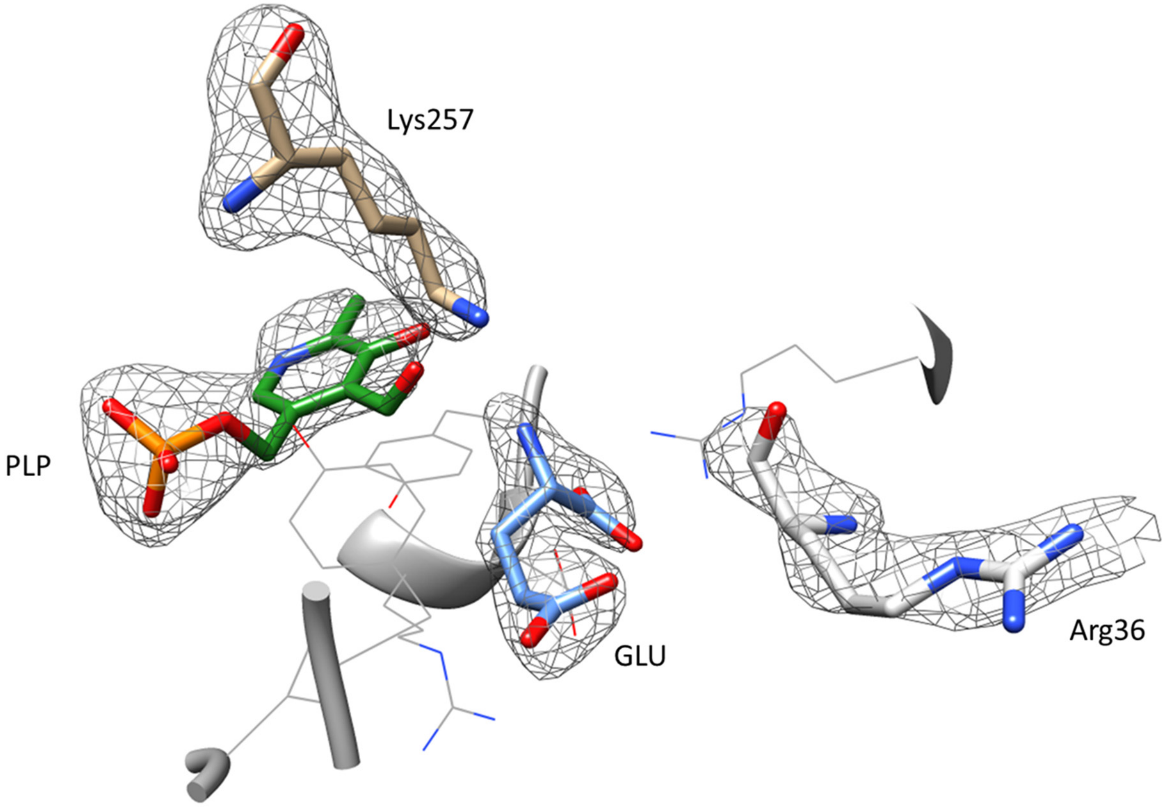

Supplementary Figure 7. Polder omit map of glutamate in the active site of Rv3722c. The catalytic Lys257 residue is shown to highlight its position relative to pyridoxal-phosphate (PLP). Arg36 does not undergo any conformational change upon binding of GLU. The map is contoured at 3σ. Other coordinating residues are shown as wire for clarity.

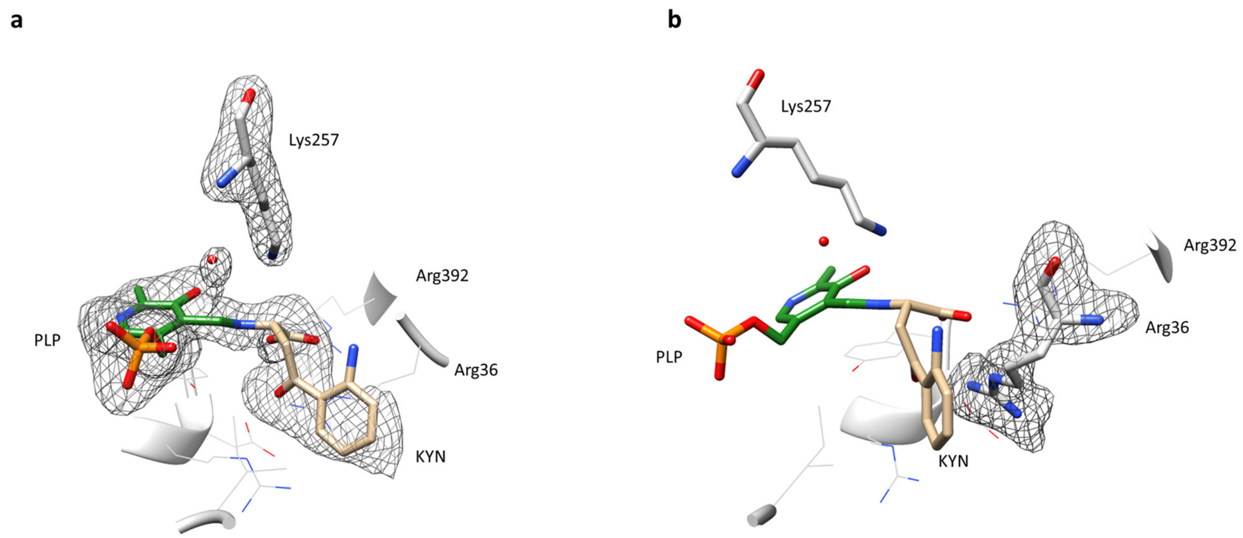

Supplementary Figure 8. Polder omit map of the PLP-Kynurenine intermediate. **A)** The catalytic Lys257 residue is shown to highlight its clear detachment from pyridoxal-phosphate (PLP). A water molecule is found to be in close proximity to both the active site Ly257 and the PLP-KYN intermediate. **B)** Upon binding KYN, Arg36 undergoes a conformational change. The maps are contoured at 4 σ. Other coordinating residues have been shown as wires for clarity. Some atoms of the arene ring of KYN did not have electron density.

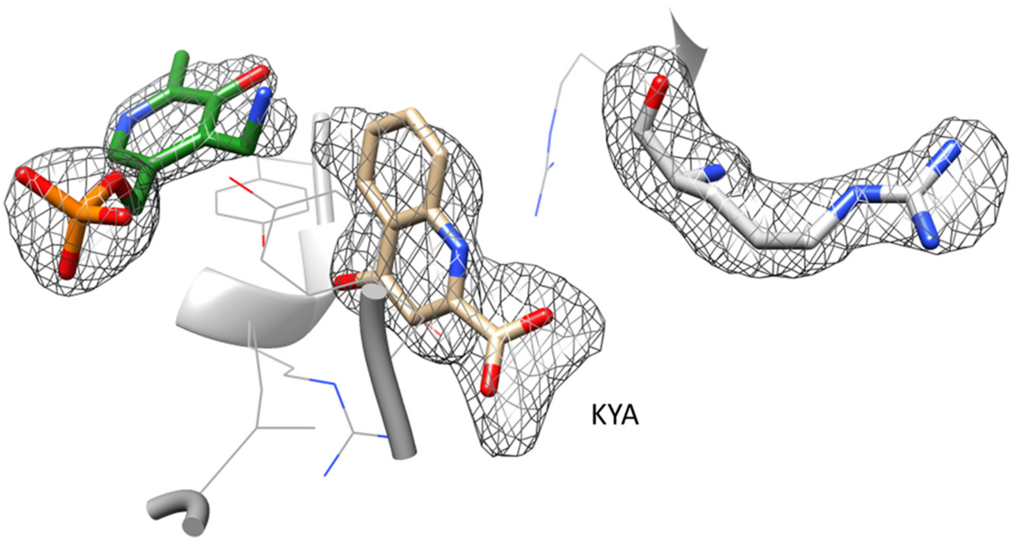

Supplementary Figure 9. Polder omit map of PMP and kynurenic acid in the active site of Rv3722c. The product kynurenic acid (KYA) adopts a different pose relative to kynurenine. Arg36 adopts its outward conformation. The map is contoured at 4 σ. For clarity, other active site residues have been displayed as wires. PMP: pyridoxamine phosphate.

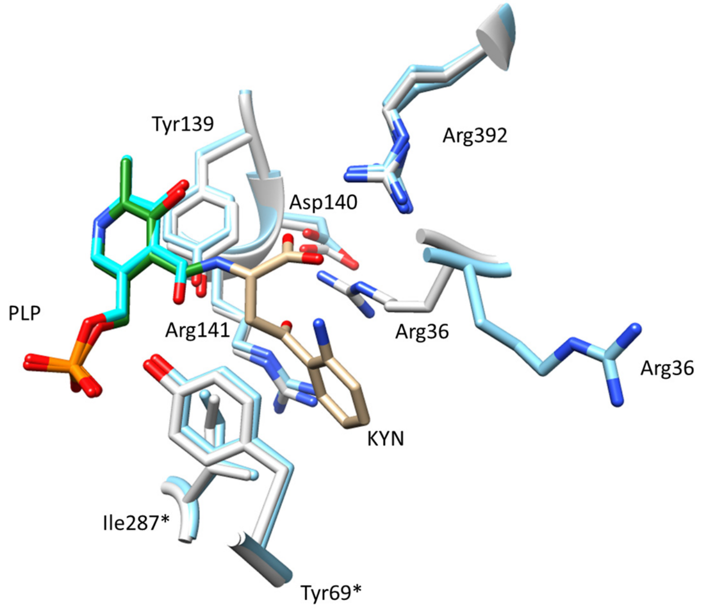

Supplementary Figure 10. Binding of kynurenine induces a conformational change. Stick and ribbon representation of the superposition of the active sites of ligand free Rv3722c (5C6U cyan) and PLP-KYN intermediate bound (grey) structures. The binding of kynurenine induces a conformational change of Arg36, resulting in the residue to move inwards to interact with the ligand.

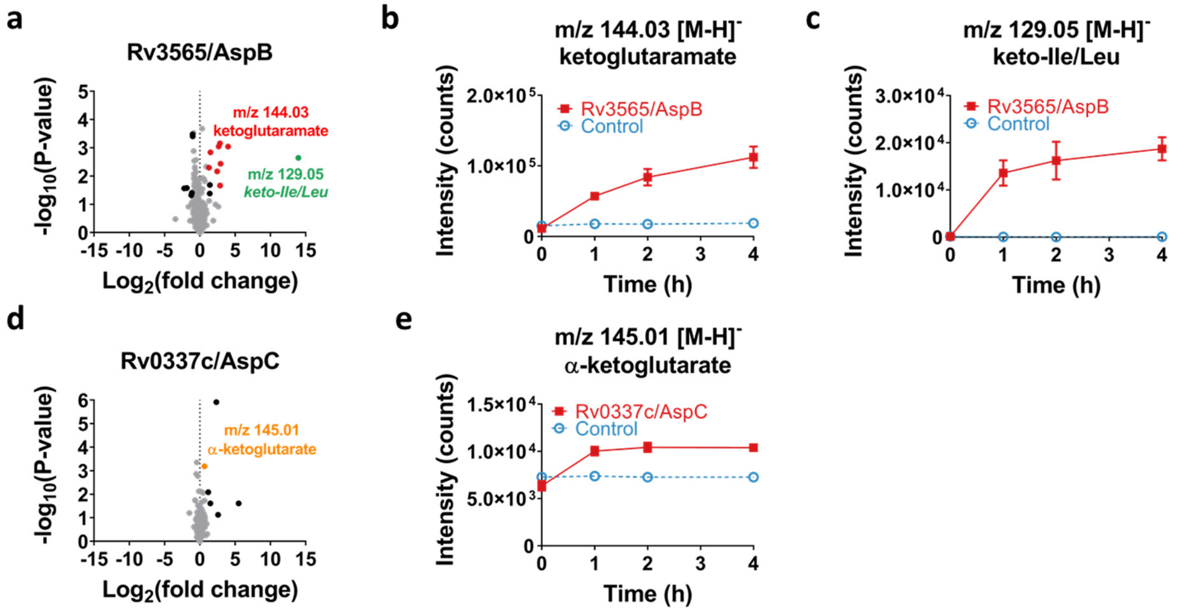

Supplementary Figure 11. Activity-based metabolite profiling on Rv3565/AspB and Rv0337c/AspC. **A)** Volcano-plot of activity-based metabolite profiling with purified recombinant Rv3565/AspB. Purified recombinant Rv3565 (10 µM) was incubated with a mycobacterial metabolite extract for 0 h or 2 h at 37 °C, and analyzed using untargeted LC-MS. Each dot represent a feature (a chromatographic peak with a specific m/z) in the negative ionization mode; red dots represent features related (fragments, adducts, dimers and isotopes) to ketoglutaramate (m/z 144.03 $[M-H]^-$), while black dots represent uncharacterized features with a fold change greater than 2 and p-value below 0.05 (n=3). **B)** Time- and Rv3565-dependent formation of ketoglutaramate. **C)** Time- and Rv3565-dependent formation of keto-Leu/Ile (m/z 129.05 $[M-H]^-$; keto-Ile/Ile could not be distinguished based on mass or retentiont time). **D)** Volcano-plot of activity-based metabolite profiling with purified recombinant Rv0337c/AspC. Same as A, but using Rv0337c/AspC incubated for 0 h or 1h at 37 °C. The orange dot represents α-ketoglutarate (m/z 145.01 $[M-H]^-$) while black dots represent uncharacterized features with a fold change greater than 2 and p-value below 0.05 (n=3). **E)** Time- and Rv0337c-dependent formation of α-ketoglutarate. Data in panel B, C and E are presented as mean +/- SD of three experimental replicates (n=3). Source data are provided as a Source Data file.

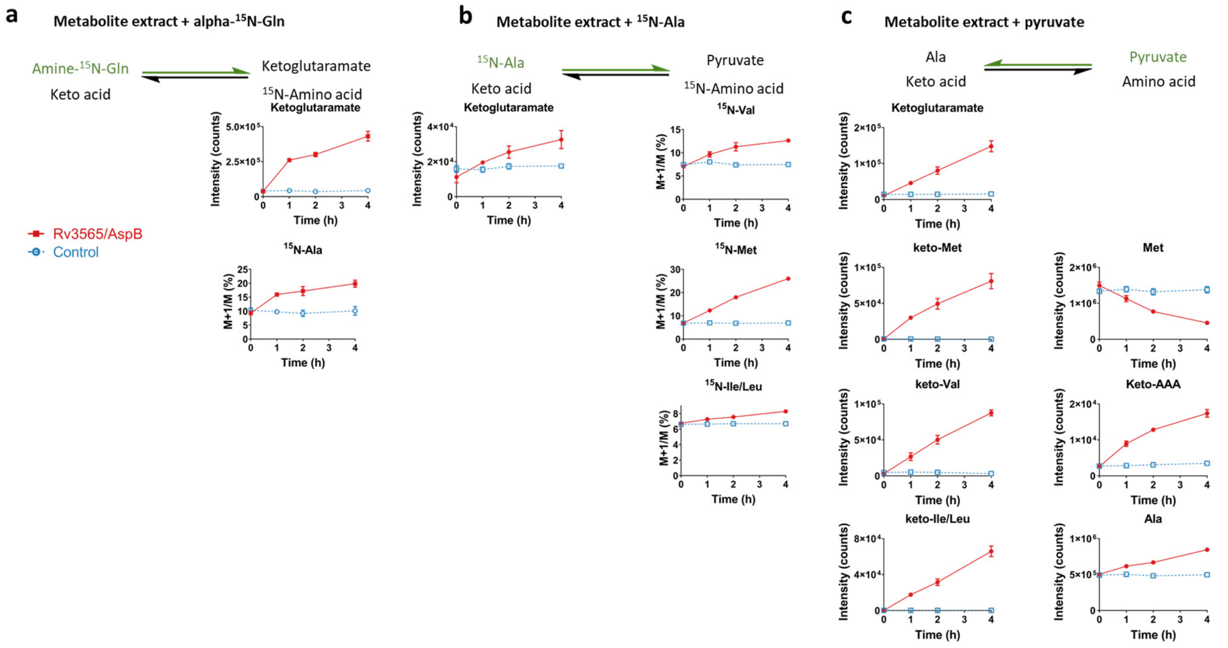

Supplementary Figure 12. Rv3565/AspB functions as an aminotransferase. **A)** Activity-based metabolite profiling with Rv3565 in the presence of α-^{15}N-Gln. Purified recombinant Rv3565 (10 µM; red line) or a buffer control, was incubated with a mycobacterial metabolite extract supplemented with 10 mM α-^{15}N-Gln for 0, 1, 2 and 4 h at 37 ^{o}C, and analyzed using untargeted LC-MS. **B)** Activity-based metabolite profiling with Rv3565 in the presence of ^{15}N-Ala. Same as A, but using a mycobacterial metabolite extract supplemented with 10 mM ^{15}N-Ala. **C)** Activity-based metabolite profiling with Rv3565 in the presence of pyruvate. Same as A, but using a mycobacterial metabolite extract supplemented with 20 mM pyruvate. Colored arrows indicate the forced direction of the Rv3565-mediated reaction. Relative metabolite levels are represented as intensity, while ^{15}N-labeling is presented as the ratio M+1/M, which was not corrected for naturally occuring isotopes. Keto-AAA, keto-Val, keto-Met, Keto-Ile/Leu: keto acids of the corresponding amino acids. Data are presented as mean +/- SD of three experimental replicates (n=3). Source data are provided as a Source Data file.

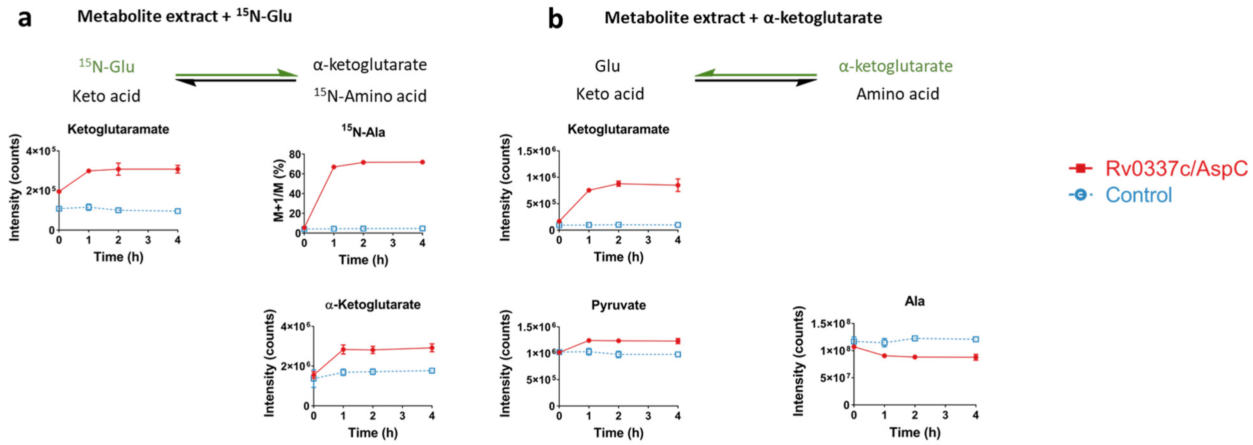

Supplementary Figure 13. Rv0337c/AspC functions as an aminotransferase. **A)** Activity-based metabolite profiling with Rv0337c in the presence of α-^{15}N-Glu. Purified recombinant Rv0337c (10 µM; red line) or a buffer control, was incubated with a mycobacterial metabolite extract supplemented with 10 mM α-^{15}N-Glu for 0, 1, 2 and 4 h at 37 ^{o}C, and analyzed using untargeted LC-MS. **B)** Activity-based metabolite profiling with Rv0337c in the presence of α-ketoglutarate. Same as A, but using a mycobacterial metabolite extract supplemented with 20 mM α-ketoglutarate. Colored arrows indicate the forced direction of the Rv0337c-mediated reaction. Relative metabolite levels are represented as intensity, while ^{15}N-labeling is presented as the ratio M+1/M, which was not corrected for naturally occuring isotopes. Data are presented as mean +/- SD of three experimental replicates (n=3). Source data are provided as a Source Data file.

a

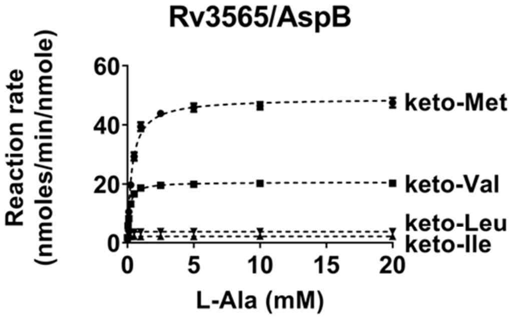

	K_M (mM)	k_{cat} (s^{-1})	k_{cat}/K_M (s^{-1} mM^{-1})
keto-Val	0.134	0.344	2.57
keto-Met	0.331	0.817	2.47

b

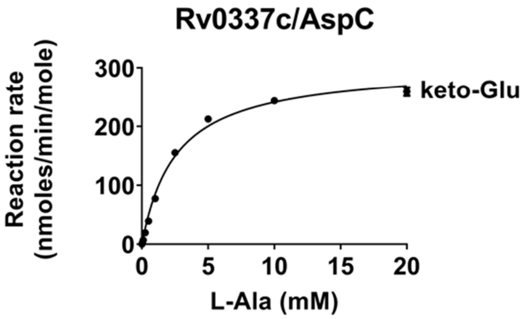

	K_M (mM)	k_{cat} (s^{-1})	k_{cat}/K_M (s^{-1} mM^{-1})
keto-Glu	2.58	5.06	1.69

Supplementary Figure 14. Rv3565/AspB functions as AvtA and Rv0337c/AspC as AlaT. **A)** Steady-state enzyme kinetics of Rv3565/AspB . Purified recombinant Rv3565 (1 µM) was incubated with 10 mM keto acids and increasing concentrations of alanine at 37 ^{o}C. Pyruvate formation was measured using a coupled reaction with lactate dehydrogenase. Measured velocities were fitted to Michaelis-Menten kinetics using Graphpad Prism 7 software. **B)** Steady-state enzyme kinetics of Rv0337c/AspC. Same as A, but for Rv0337c. Data are presented as mean +/- SD of three experimental replicates (n=3). Source data are provided as a Source Data file.

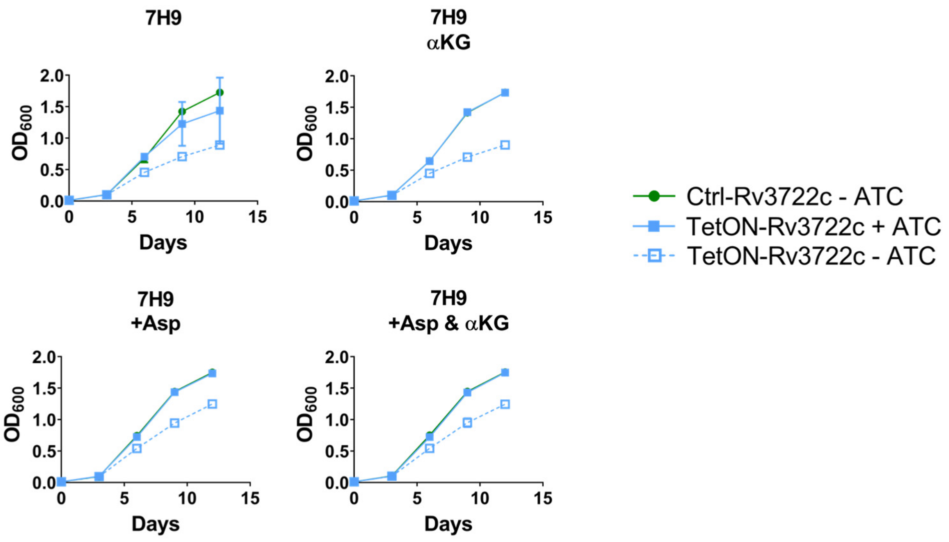

Figure 15. Alpha-ketoglutarate supplementation does not rescues growth of Rv3722c-deficient *Mtb*. Growth curve of Rv3722c-proficient and –deficient *Mtb* in 7H9 culture media with or without 3 mM alpha-ketoglutarate and/or 3 mM aspartate. Rv3722c-TetOn and Rv3722c-control were cultured in Middlebrook 7H9 culture media with or without 500 ng mL^{-1} anhydrotetracycline (ATC). Bacterial growth was monitored by optical density at 600 nm. For all growth curves, data are represented as mean -/+ SD of three experimental replicates (n=3). Source data are provided as a Source Data file.

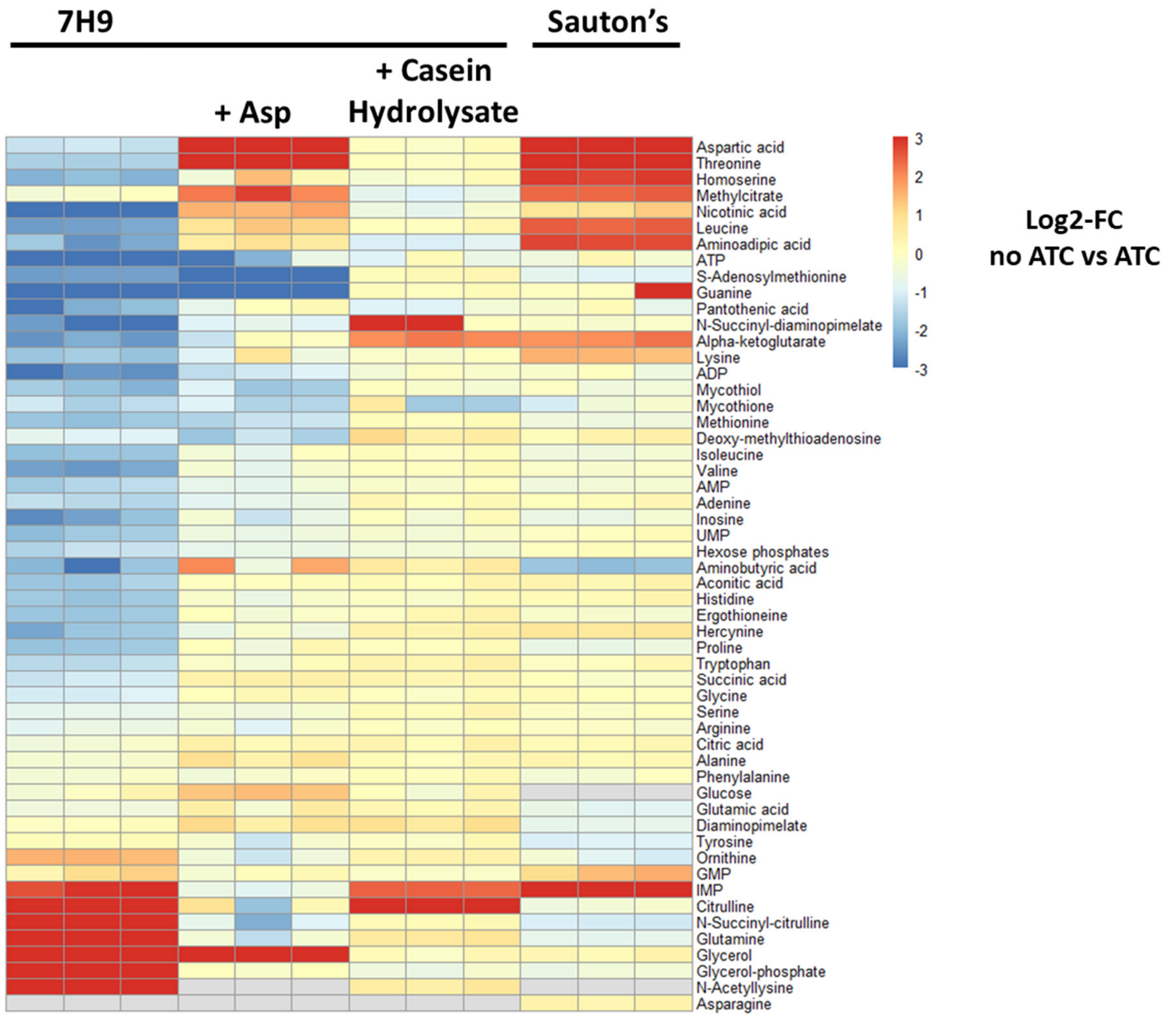

Supplementary Figure 16. Rv3722c deficiency results in widespread metabolic changes. Heatmap showing the log2-fold change in metabolite levels in growth permissive and non-permissive culture media (Fig 1A, C, E and 7B). Rv3722c-TetON and Rv3722c-control, were cultured in 7H9, 7H9+3 mM aspartate, 7H9+ 1% casein hydrolysate (all with 0.04% tyloxapol), and Sauton's minmal media, in the presence and absence of 500 ng mL^{-1} ATC. After 12 days, metabolite levels were obtained using high-resolution LC-MS. Colors represent log2-fold change of Rv3722c-deficient (no ATC) versus –proficient (ATC) Rv3772-TetON. Data are presented as mean ratio of three experimental replicates (n=3 vs n=3). Source data are provided as a Source Data file.

Supplementary References

1. Adams, P. D. *et al.* PHENIX: building new software for automated crystallographic structure determination. *Acta Crystallogr. D Biol. Crystallogr.* **58**, 1948–1954 (2002).
2. Zwart, P. H., Grosse-Kunstleve, R. W., Lebedev, A. A., Murshudov, G. N. & Adams, P. D. Surprises and pitfalls arising from (pseudo)symmetry. *Acta Crystallogr. D Biol. Crystallogr.* **64**, 99–107 (2008).
3. Brooks, C. L. *et al.* Pseudo-symmetry and twinning in crystals of homologous antibody Fv fragments. *Acta Crystallogr. D Biol. Crystallogr.* **64**, 1250–1258 (2008).
4. Son, H. F. & Kim, K.-J. Structural Insights into a Novel Class of Aspartate Aminotransferase from Corynebacterium glutamicum. *PloS One* **11**, e0158402 (2016).
5. Gibrat, J. F., Madej, T. & Bryant, S. H. Surprising similarities in structure comparison. *Curr. Opin. Struct. Biol.* **6**, 377–385 (1996).
6. Dolzan, M. *et al.* Crystal structure and reactivity of YbdL from Escherichia coli identify a methionine aminotransferase function. *FEBS Lett.* **571**, 141–146 (2004).
7. Mehta, P. K., Hale, T. I. & Christen, P. Aminotransferases: demonstration of homology and division into evolutionary subgroups. *Eur. J. Biochem.* **214**, 549–561 (1993).
8. Dolzan, M. *et al.* Crystal structure and reactivity of YbdL from Escherichia coli identify a methionine aminotransferase function. *FEBS Lett.* **571**, 141–146 (2004).
9. Liebschner, D. *et al.* Polder maps: improving OMIT maps by excluding bulk solvent. *Acta Crystallogr. Sect. Struct. Biol.* **73**, 148–157 (2017).
10. Malashkevich, V. N., Onuffer, J. J., Kirsch, J. F. & Jansonius, J. N. Alternating arginine-modulated substrate specificity in an engineered tyrosine aminotransferase. *Nat. Struct. Biol.* **2**, 548–553 (1995).
11. Eliot, A. C. & Kirsch, J. F. Pyridoxal phosphate enzymes: mechanistic, structural, and evolutionary considerations. *Annu. Rev. Biochem.* **73**, 383–415 (2004).
