# Supplementary material for: Aspartate aminotransferase Rv3722c governs aspartate-dependent nitrogen metabolism in Mycobacterium tuberculosis
Source: Nat Commun. 2020 Apr 23;11:1960. doi: 10.1038/s41467-020-15876-8 (PMC7181641; doi:10.1038/s41467-020-15876-8)
Supplement: Supplementary file 3 — Reporting Summary [file 41467_2020_15876_MOESM3_ESM.pdf]

nature research

Corresponding author(s): Kyu Rhee and James Sacchettini

Last updated by author(s): Mar 25, 2020

Reporting Summary

Nature Research wishes to improve the reproducibility of the work that we publish. This form provides structure for consistency and transparency in reporting. For further information on Nature Research policies, see Authors & Referees and the Editorial Policy Checklist .

Statistics

For all statistical analyses, confirm that the following items are present in the figure legend, table legend, main text, or Methods section.

n/a	Confirmed	
☐	☒	The exact sample size (*n*) for each experimental group/condition, given as a discrete number and unit of measurement
☐	☒	A statement on whether measurements were taken from distinct samples or whether the same sample was measured repeatedly
☐	☒	The statistical test(s) used AND whether they are one- or two-sided<br>*Only common tests should be described solely by name; describe more complex techniques in the Methods section.*
☒	☐	A description of all covariates tested
☒	☐	A description of any assumptions or corrections, such as tests of normality and adjustment for multiple comparisons
☐	☒	A full description of the statistical parameters including central tendency (e.g. means) or other basic estimates (e.g. regression coefficient) AND variation (e.g. standard deviation) or associated estimates of uncertainty (e.g. confidence intervals)
☒	☐	For null hypothesis testing, the test statistic (e.g. *F*, *t*, *r*) with confidence intervals, effect sizes, degrees of freedom and *P* value noted<br>*Give P values as exact values whenever suitable.*
☒	☐	For Bayesian analysis, information on the choice of priors and Markov chain Monte Carlo settings
☒	☐	For hierarchical and complex designs, identification of the appropriate level for tests and full reporting of outcomes
☒	☐	Estimates of effect sizes (e.g. Cohen's *d*, Pearson's *r*), indicating how they were calculated

Our web collection on statistics for biologists contains articles on many of the points above.

Software and code

Policy information about availability of computer code

Data collection

- Masshunter Workstation Software - Data Acquisition (Version B.06.01/Build 6.01.6157; Agilent)
- Masshunter Workstation Software - Data Acquisition (Version B.08.00/Build 8.00.8058.0; Agilent)
- Masshunter Workstation Software - Data Acquisition (Version B.01.01/Build 7.1.7112.4/SP1; Agilent)
- RapidFire Software (Version 4.0.12333.14247; Agilent)
- Image Studio (Version 5.2; LI-COR Biosciences)

Data analysis	- Masshunter Workstation Software - Qualitative Analysis Version (B.07.00/SP2; Agilent)<br>- Masshunter Workstation Software – Profinder (B.08.00/SP3; Agilent)<br>- Proteowizard (Version 3.0.7162)<br>- XCMS online (Version 3.5.1)<br>- Pheatmap R package (Version 1.0.12)<br>- Excel 2016 (Microsoft)<br>- Masshunter Workstation Software – Quantitative Analysis (B.07.00/Build 7.0.457.0; Agilent)<br>- Graphpad prism (Version 8.0.0)<br>- Image Studio Lite (Version 5.2; LI-COR Biosciences)<br>- Proteum3 (Version 2016.2)<br>- CCP4 suite (Version 7.0.072)<br>- XDS (Version Mar 15, 2019, Built=20190315)<br>- STARANISO webserver (OpenMP version 2.3.9 (5-Aug-2019))<br>- PHENIX (Version 1.17.1-3660)<br>- COOT (Version 0.8.8)<br>- Chimera (Version 1.13.1)

For manuscripts utilizing custom algorithms or software that are central to the research but not yet described in published literature, software must be made available to editors/reviewers. We strongly encourage code deposition in a community repository (e.g. GitHub). See the Nature Research guidelines for submitting code & software for further information.

Data

Policy information about availability of data

All manuscripts must include a data availability statement. This statement should provide the following information, where applicable:
- Accession codes, unique identifiers, or web links for publicly available datasets
- A list of figures that have associated raw data
- A description of any restrictions on data availability

Structural data that support the findings of this study have been deposited in the Protein Data Bank with the accession codes 6U78 [https://www.rcsb.org/structure/6U78] (Rv3722/Glu) and 6U7A [https://www.rcsb.org/structure/6U7A] (Rv3722/KYN). The source data underlying Figs 1a-c, e, f; 2a–b; 3a, b, d; 5a, b; 6; 7a-e; 8 a-b; and Supplementary Figures 1a,b; 2; 3a,b; 4a-c; 5; 11a-e; 12a-c; 13a,b; 14a,b; 15 and 16 are provided as a Source Data file. Metabolomics datasets that support the main findings of this study are available from the Metabolights repository with the accession code MTBLS1559 [www.ebi.ac.uk/metabolights/MTBLS1559]. The data underlying the phylogenetic tree for PF12897 was downloaded from Annotree [http://annotree.uwaterloo.ca/] (AnnoTree v1.1.0; GTDB Bacteria Release 03-RS86; Pfam v27.0; E-value of 0.00001).

Field-specific reporting

Please select the one below that is the best fit for your research. If you are not sure, read the appropriate sections before making your selection.

☒ Life sciences ☐ Behavioural & social sciences ☐ Ecological, evolutionary & environmental sciences

For a reference copy of the document with all sections, see nature.com/documents/nr-reporting-summary-flat.pdf

Life sciences study design

All studies must disclose on these points even when the disclosure is negative.

Sample size	For mouse experiments, n=5 based on power calculations to detect a difference of at least 1 log10 in CFU between two strains (a= 0.05, power = 95%), in lungs and spleens. For all other experiments, n=3 based on the use of biologically independent within-experiment triplicates to allow determination of standard errors, and biological validation by replication in at least 2 independent experiments.
Data exclusions	In Figure 8a single value for succinyl-DAP (15N-Asp +ATC) is left out because of an interfering peak with the mass of M+2 succinyl-DAP. This exclusion criterium was not pre-established.
Replication	All findings described in the manuscript were found to be reproducible.
Randomization	Mice were randomly assigned to experimental groups.
Blinding	Blinding was not required for the described experiments, because they did not involve subjective scoring.

Reporting for specific materials, systems and methods

We require information from authors about some types of materials, experimental systems and methods used in many studies. Here, indicate whether each material, system or method listed is relevant to your study. If you are not sure if a list item applies to your research, read the appropriate section before selecting a response.

Materials & experimental systems

n/a	Involved in the study	
☐	☒	Antibodies
☒	☐	Eukaryotic cell lines
☒	☐	Palaeontology
☐	☒	Animals and other organisms
☒	☐	Human research participants
☒	☐	Clinical data

Methods

n/a	Involved in the study	
☒	☐	ChIP-seq
☒	☐	Flow cytometry
☒	☐	MRI-based neuroimaging

Antibodies

Antibodies used	Monoclonal mouse anti-FLAG antibody M2 (Sigma F3165), Clone M2, Lot SLBQ7119V<br>Rabbit anti-prcB (a gift from G. Lin and C. Nathan, who generated this non-commercially available antiserum, GN-13.782 3#; stock 1-76)<br>IRDye 800CW Goat Anti-Mouse IgG (H+L) (LI-COR Biosciences)<br>IRDye® 680LT Donkey anti-Rabbit IgG (H + L) (LI-COR Biosciences)
Validation	The mouse anti-FLAG antibody is a commercially available and widely used antibody for immunoblotting analysis (Sigmaaldrich.com), including in Mycobacterium tuberculosis (PLoS One. 2011; e21738). The rabbit anti-prcB antibody is specific (Nat Med. 2007; 13(12): 1515–1520), and has been used extensively as a loading control for immunoblotting in mycobacteria (see for example Cell Chem Biol. 2017; 24(3):306-315, Nucleic Acids Res. 2011; 39(6): 2210–2220, and EMBO J. 2006; 25 (22):5423-32.)

Animals and other organisms

Policy information about studies involving animals; ARRIVE guidelines recommended for reporting animal research

Laboratory animals	Femoral mouse bone marrow cells were isolated from 7-8 week old, male C57BL/6J mice.<br>Mouse infection were performed on 6-8 week old female BL/6 mice. Mice were housed at 19-23 degrees Celsius, a humidity from 35-60% and a 14h/10h light/dark cycle.
Wild animals	The study did not involve wild animals.
Field-collected samples	The study did not involve samples collected from the field.
Ethics oversight	Oversight for the collection of primary bone-marrow derived macrophages was provided by the Institutional Animal Care and Use Committee of Weill Cornell Medicine.<br>For the mouse infections, oversight was provided by the Harvard Medical Area Institutional Animal Care and Use Committee.

Note that full information on the approval of the study protocol must also be provided in the manuscript.
